# Supplementary material for: Multi‐dataset identification of innovative feature genes and molecular mechanisms in keratoconus
Source: J Cell Mol Med. 2024 Sep 19;28(18):e70079. doi: 10.1111/jcmm.70079 (PMC11412914; doi:10.1111/jcmm.70079)
Supplement: Supplementary file 2 — Material S2. [file JCMM-28-e70079-s002.zip › 7.GSEA analysis/2.ATOH7/KEGG_GLYCOSAMINOGLYCAN_BIOSYNTHESIS_HEPARAN_SULFATE.html]

Details for gene set KEGG\_GLYCOSAMINOGLYCAN\_BIOSYNTHESIS\_HEPARAN\_SULFATE[GSEA]

|  || Dataset | ATOH7.group.cls#h\_versus\_l |
| Phenotype | group.cls#h\_versus\_l |
| Upregulated in class | l |
| GeneSet | KEGG\_GLYCOSAMINOGLYCAN\_BIOSYNTHESIS\_HEPARAN\_SULFATE |
| Enrichment Score (ES) | -0.2668074 |
| Normalized Enrichment Score (NES) | -0.8883546 |
| Nominal p-value | 0.6081081 |
| FDR q-value | 1.0 |
| FWER p-Value | 1.0 |
Table: GSEA Results Summary

  

Fig 1: Enrichment plot: KEGG\_GLYCOSAMINOGLYCAN\_BIOSYNTHESIS\_HEPARAN\_SULFATE      
 Profile of the Running ES Score & Positions of GeneSet Members on the Rank Ordered List

  

| SYMBOL | TITLE | RANK IN GENE LIST | RANK METRIC SCORE | RUNNING ES | CORE ENRICHMENT || 1 | NDST4 | na | 1792 | 0.360 | 0.0703 | No |
| 2 | HS3ST5 | na | 4025 | 0.236 | 0.0857 | No |
| 3 | GLCE | na | 5372 | 0.189 | 0.1110 | No |
| 4 | B3GAT2 | na | 7780 | 0.123 | 0.0833 | No |
| 5 | EXTL2 | na | 8537 | 0.107 | 0.0979 | No |
| 6 | B4GALT7 | na | 9120 | 0.097 | 0.1139 | No |
| 7 | HS2ST1 | na | 9702 | 0.086 | 0.1263 | No |
| 8 | EXT2 | na | 10394 | 0.075 | 0.1318 | No |
| 9 | EXT1 | na | 10919 | 0.067 | 0.1394 | No |
| 10 | B3GAT1 | na | 11158 | 0.064 | 0.1541 | No |
| 11 | EXTL1 | na | 12058 | 0.052 | 0.1458 | No |
| 12 | XYLT2 | na | 13122 | 0.039 | 0.1284 | No |
| 13 | B3GAT3 | na | 13394 | 0.036 | 0.1328 | No |
| 14 | NDST2 | na | 15800 | 0.014 | 0.0684 | No |
| 15 | XYLT1 | na | 16288 | 0.010 | 0.0579 | No |
| 16 | B3GALT6 | na | 16308 | 0.010 | 0.0609 | No |
| 17 | HS3ST2 | na | 16516 | 0.009 | 0.0578 | No |
| 18 | EXTL3 | na | 17775 | -0.002 | 0.0222 | No |
| 19 | HS6ST2 | na | 18358 | -0.007 | 0.0079 | No |
| 20 | HS6ST1 | na | 21992 | -0.043 | -0.0823 | No |
| 21 | HS3ST1 | na | 22023 | -0.043 | -0.0686 | No |
| 22 | NDST3 | na | 28903 | -0.176 | -0.2073 | Yes |
| 23 | NDST1 | na | 30429 | -0.226 | -0.1747 | Yes |
| 24 | HS3ST3B1 | na | 31586 | -0.273 | -0.1156 | Yes |
| 25 | HS6ST3 | na | 31939 | -0.290 | -0.0276 | Yes |
| 26 | HS3ST3A1 | na | 32386 | -0.319 | 0.0675 | Yes |
Table: GSEA details [plain text format]

  

Fig 2: KEGG\_GLYCOSAMINOGLYCAN\_BIOSYNTHESIS\_HEPARAN\_SULFATE      
 Blue-Pink O' Gram in the Space of the Analyzed GeneSet

  

Fig 3: KEGG\_GLYCOSAMINOGLYCAN\_BIOSYNTHESIS\_HEPARAN\_SULFATE: Random ES distribution      
 Gene set null distribution of ES for **KEGG\_GLYCOSAMINOGLYCAN\_BIOSYNTHESIS\_HEPARAN\_SULFATE**
